# Phytochemical Composition and Bioactivities of Some Hydrophytes: Antioxidant, Antiparasitic, Antibacterial, and Anticancer Properties and Mechanisms

**DOI:** 10.3390/plants13152148

**Published:** 2024-08-02

**Authors:** Fahad Alharthi, Hussam A. Althagafi, Ibrahim Jafri, Atif Abdulwahab A. Oyouni, Mohammed M. Althaqafi, Layla Yousif Abdullah Al-Hijab, Nawal E. Al-Hazmi, Somia M. Elagib, Deyala M. Naguib

**Affiliations:** 1 Department of Biology, College of Science, Taif University, P.O. Box 11099, Taif 21944, Saudi Arabia; f.alharthi@tu.edu.sa; 2 Department of Biology, Faculty of Science, Al-Baha University, Al-Baha 65525, Saudi Arabia; halthaqafi@bu.edu.sa (H.A.A.); lalhejab@bu.edu.sa (L.Y.A.A.-H.); smmohamed@bu.edu.sa (S.M.E.); 3 Department of Biotechnology, College of Science, Taif University, P.O. Box 11099, Taif 21944, Saudi Arabia; i.jafri@tu.edu.sa (I.J.); mm.mohammad@tu.edu.sa (M.M.A.); 4 Department of Biology, Faculty of Science, University of Tabuk, Tabuk 71491, Saudi Arabia; a.oyouni@ut.edu.sa; 5 Biodiversity Genomics Unit, Faculty of Science, University of Tabuk, Tabuk 71491, Saudi Arabia; 6 Department of Chemistry, Division of Biology (Microbiology), University College of Qunfudah, Umm Al-Qura University, Qunfudah 21961, Saudi Arabia; nehazmi@uqu.edu.sa; 7 Science Department, Faculty of Teachers, Nile Valley University, Edammer, Atbara 46611, Sudan; 8 Botany and Microbiology Department, Faculty of Science, Zagazig University, Zagazig 44511, Egypt

**Keywords:** apoptosis, biofilm inhibition, bacterial DNA, carcinogenic bacteria, cytochrome c release, flavonoids, liver flukes, mitochondrial membrane permeability, phenols

## Abstract

Few researches have explored the production of pharmaceuticals from aquatic plants. Therefore, this study explored, for the first time, the phytochemical composition and bioactivities of ten aquatic plants. Aquatic plant shoots from various Nile River canals were collected, dried, and ground for aqueous extract preparation. Phytochemical composition and antioxidant capacity were assessed using DPPH assays. Extracts were tested for antiparasitic, antibacterial, anti-biofilm, and anticancer activities through standard in vitro assays, measuring IC_50_ values, and evaluating mechanisms of action, including cell viability and high-content screening assays. The results showed that the aquatic plants were rich in pharmaceutical compounds. The antioxidant capacity of these extracts exceeded that of vitamin C. The extracts showed promising antiparasitic activity against pathogens like *Opisthorchis viverrini* and *Plasmodium falciparum*, with IC_50_ values between 0.7 and 2.5 µg/mL. They also demonstrated low MICs against various pathogenic bacteria, causing DNA damage, increased plasma membrane permeability, and 90% biofilm inhibition. In terms of anticancer activity, extracts were effective against a panel of cancer cell lines, with *Ludwigia stolonifera* exhibiting the highest efficacy. Its IC_50_ ranged from 0.5 µg/mL for pancreatic, esophageal, and colon cancer cells to 1.5 µg/mL for gastric cancer cells. Overall, IC_50_ values for all extracts were below 6 µg/mL, showing significant apoptotic activity, increased nuclear intensity, plasma membrane permeability, mitochondrial membrane permeability, and cytochrome c release, and outperforming doxorubicin. This study highlights the potential of aquatic plants as sources for new, safe, and effective drugs with strong antiparasitic, antibacterial, and anticancer properties.

## 1. Introduction

Pathogens continually evolve resistance to existing pharmaceuticals, rendering them ineffective. Discovering new compounds can provide alternative treatment options to combat drug-resistant strains of bacteria, viruses, and parasites [1,2,3]. In addition, there are unmet medical needs for various diseases, including cancer, neurodegenerative disorders, and neglected tropical diseases. Exploring new natural compounds offers the potential to develop treatments for these conditions, improving global healthcare. Therefore, exploring new natural, safe pharmaceutical compounds is crucial [4,5,6,7,8,9].

Natural compounds often have a long history of safe use in traditional medicine. Studying these compounds can lead to the development of pharmaceuticals with fewer adverse effects compared to synthetic drugs, which can have detrimental adverse effects on human health. By harnessing natural compounds, sourced sustainably from plants, fungi, and marine organisms, we can develop pharmaceuticals with lower environmental impact [10,11,12]. Earth’s biodiversity is a vast resource for potential pharmaceutical compounds. Exploring diverse ecosystems allows us to discover novel molecules with therapeutic potential, expanding the pharmacological toolkit available to medical professionals [13,14,15,16]. Natural compounds often possess unique chemical structures and biological activities, providing opportunities for drug discovery and innovation. These compounds may target disease pathways not addressed by existing pharmaceuticals, leading to breakthrough treatments [17,18]. The antioxidant activity of plants is the main biological activity that gives plants medicinal importance [19]. The antioxidant activity of plants is related to their chemical constituents [20]. Phenols are the most common plant constituents with antioxidant activity [21].

Aquatic plants have long been recognized as potential sources of pharmaceutical compounds, due to their rich biodiversity and the unique chemical constituents they produce. Researchers have been exploring aquatic plants for bioactive compounds with medicinal properties [22,23,24]. Water hyacinth has been reported to have pharmaceutical components with promising antioxidant, antimicrobial, and anticancer activity [25,26,27,28]. Lotus (*Nelumbo nucifera*) is another common aquatic plant that has high biologically active compounds such as phenols, flavonoids, and alkaloids, which have shown promise for medicinal application [29]. Water celery (*Oenanthe javanica*) produces compounds with anti-inflammatory properties, which can be useful in the treatment of inflammatory conditions such as arthritis and asthma [30,31]. Water mint (*Mentha aquatica*) showed promising phytochemical components with high antioxidant activity and that were efficiently used against skin cancer [32,33]. Researchers have identified anticancer compounds from watercress (*Nasturtium officinale*) that showed a promising effect in inhibiting the growth of cancer cells or inducing apoptosis [34,35]. Overall, the diverse array of bioactive compounds found in aquatic plants offers great potential for the development of novel pharmaceuticals and therapeutic agents. However, further research is necessary to fully explore and harness the medicinal properties of these plants [23,36]. From this point of view, we tried in this work to investigate the phytochemical components and diverse biological activities (antioxidant, antiparasitic, antibacterial, and anticancer activities) of some aquatic plants. We selected aquatic plants that have not been thoroughly studied for their phytochemical components and biological activity (Supplementary data Table S1, Figure S1). *Scirpus maritimus* is a perennial, grass-like plant with erect stems and a clump-forming habit, belonging to the Cyperaceae family. *Lemna gibba* (Gibbous Duckweed) belongs to the Araceae family and is a small, free-floating aquatic plant with a single leaf (frond) and a root hanging below. It often forms dense mats on the surface of still or slow moving freshwater. *Ottelia alismoides* (Duck Lettuce) belongs to the Hydrocharitaceae family and is an aquatic plant with submerged or floating leaves. The leaves are broad and often have a distinctive pattern of veins, and it produces small, white or yellowish flowers. *Ruppia maritima* (Widgeon Grass) is a submerged, thread-like aquatic plant with slender stems and narrow leaves, belonging to the Ruppiaceae family. It is highly tolerant of salinity and often found in brackish or saline waters. *Zannichellia palustris* (Horned Pondweed) belongs to the *Zannichelliaceae* family and is a submerged aquatic plant with thin, branched stems and narrow, linear leaves. It has small, inconspicuous flowers. *Ludwigia stolonifera*, belonging to the Onagraceae family, is a creeping or trailing perennial plant with small, yellow flowers and green, oval leaves. It can grow both submerged and emergent. *Alisma plantago-aquatica* (Water Plantain) belongs to the Alismataceae family and is a perennial herb with broad, lance-shaped leaves and small white or pink flowers arranged in whorls. It grows in shallow water or wet soil. *Damasonium alisma* (Starfruit) also belongs to the Alismataceae family and is an annual or perennial aquatic plant with rosette-forming leaves and star-shaped white flowers. It produces distinctive star-shaped fruits. *Carex divisa* (Divided Sedge) belongs to the Cyperaceae family and is a perennial sedge with clump-forming, tufted growth and narrow, grass-like leaves. It produces clusters of small, greenish-brown flower spikes. *Leptochloa fusca* (Sprangletop) belongs to the Poaceae family and is a perennial grass with a spreading growth habit and long, narrow leaves. It produces open, airy flower panicles [37].

## 2. Results and Discussion

### 2.1. Phytochemical Components and Antioxidant Capacity of the Extracts

**Figure 1:**
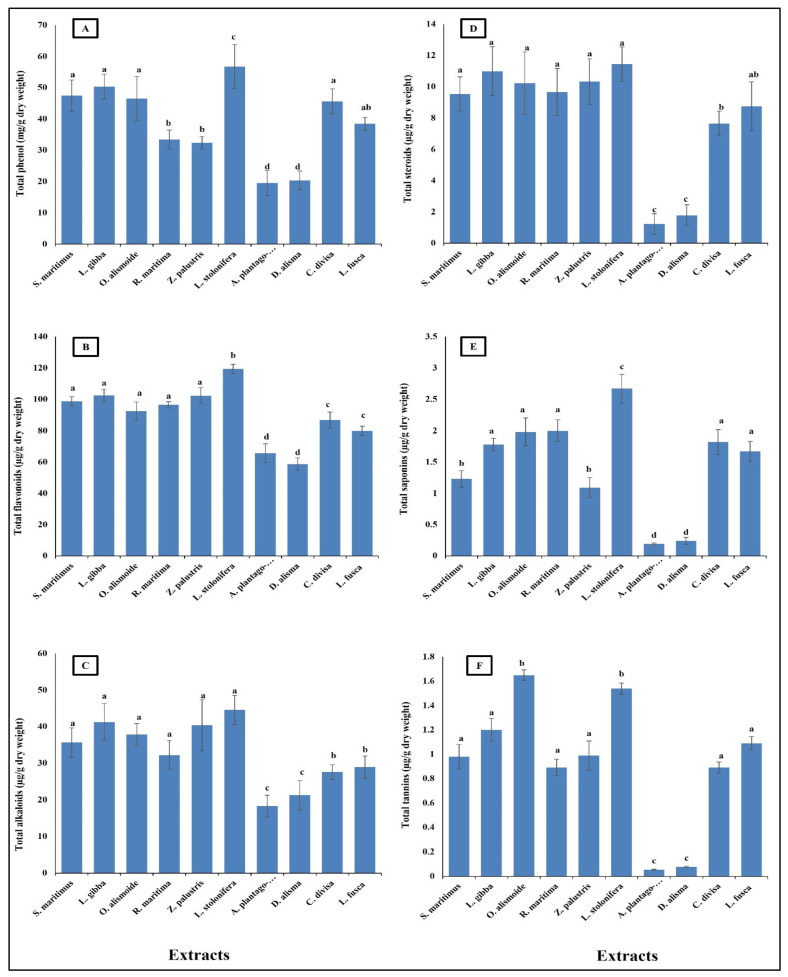
Phytochemical component content [(**A**) total phenols (mg/g dry wt), (**B**) flavonoid, (**C**) alkaloid, (**D**) steroid, (**E**) saponin, (**F**) tannin (µg/g dry wt)] of different aquatic plants extracts. Column value is the mean of five replicates. The error bars represent standard deviation. Columns followed with different letters are significantly different according to ANOVA test.

Aquatic plants have long been recognized as potential sources of pharmaceutical compounds, due to their rich biodiversity and the unique chemical constituents they produce [38,39,40]. The present results showed that the aquatic plants were rich in pharmaceutical compounds (Figure 1). Phenols showed to be the most abundant phytochemicals. *Ludwigia stolonifera* had the highest content of phytochemical components, followed by *Scirpus maritimus*, *Lemna gibba*, *Otellia alismoide*, *Ruppia maritima*, *Zannichellia palustris*, *Carex divisa*, and *Leptochloa fusca*. The lowest content of phytochemicals was recorded in *Alisma plantago-aquatica* and *Damasonium alisma*. Aquatic plants contain a diverse array of secondary metabolites. Each compound plays a specific role in the plant’s ecology and physiology, contributing to its adaptation to the aquatic environment [41,42,43]. Phenolic compounds are common secondary metabolites in aquatic plants. They have antioxidant properties and can help protect the plant from oxidative stress. Tannins are polyphenol compounds known for their astringent properties. They are commonly found in various parts of aquatic plants, including leaves, stems, and roots [44,45]. Tannins can serve as chemical defenses against herbivores by making plant tissues unpalatable or toxic [46,47]. Some aquatic plants produce alkaloids, nitrogen-containing compounds with various biological activities. Alkaloids can act as toxins to deter herbivores or inhibit the growth of competing plants. Saponins are glycosides with foaming properties. They can act as natural surfactants and have been found in some aquatic plants [48,49]. Saponins play a role in defense against different stresses [50]. Steroids are a class of lipids with a characteristic structure consisting of four fused rings. While steroids are more commonly associated with animals, some aquatic plants also produce steroid compounds. Phytosterols, which are plant-derived steroids, are important components of cell membranes in aquatic plants. These compounds help maintain membrane integrity and fluidity, which is crucial for various cellular processes. Phytosterols also have antioxidant properties, protecting cells from oxidative damage caused by reactive oxygen species [51,52,53].

**Figure 2:**
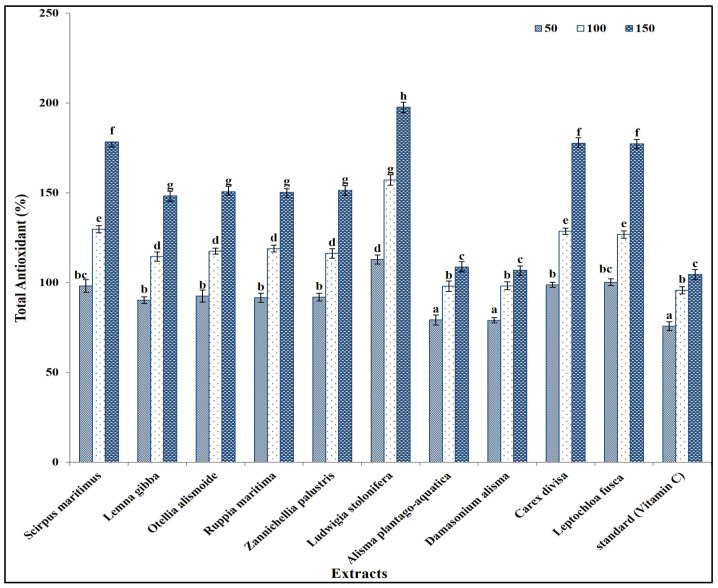
Antioxidant capacity of different concentrations (50, 100, and 150 mg/mL) of prepared extracts in comparison with standard (Vitamin C). Column value is the mean of five replicates. The error bars represent standard deviation. Columns followed with different letters are significantly different according to ANOVA test.

These secondary metabolites are known to contribute to the antioxidant activity of plant extracts, due to their ability to scavenge free radicals and inhibit oxidative processes [54]. The total antioxidant capacity of the prepared extracts was in the same line as the results of the phytochemical components in extracts, as *L. stolonifera* had the highest antioxidant capacity, followed by *C. divisa*, *L. fusca*, *S. maritimus*, *L. gibba*, *O. alismoide*, *R. maritima*, and *Z. palustris*. The lowest antioxidant capacity was recorded in *A. plantago-aquatica* and *D. alisma* (Figure 2).

This is in the same line as Al-Rowaily et al. [55], who reported that antioxidants increased with increasing phytochemical components of certain geophyte sedges and grasses. In addition, Alzandi et al. [56] reported a positive correlation between the antioxidant capacity of a plant extract and its phytochemical component content. Similarly, Kochar et al. [57] reported that antioxidant capacity was related to the secondary metabolites content in a plant extract. The increase in secondary metabolite content in a plant extract correlated with the increase in antioxidant capacity, due to the higher concentration, greater diversity, synergistic interactions, efficient free radical scavenging, and enhanced stability of antioxidants present in the extract [54].

Interestingly, the antioxidant capacity of all prepared extracts was significantly higher than that of vitamin C (the standard) (Figure 2). This is because the antioxidant capacity of plant extracts is often influenced by the combined action of multiple secondary metabolites. Different classes of secondary metabolites may interact synergistically, enhancing the overall antioxidant activity beyond what would be expected based on the individual contributions of each compound [58,59].

### 2.2. Antiparasitic Activity of the Extracts

**Figure 3:**
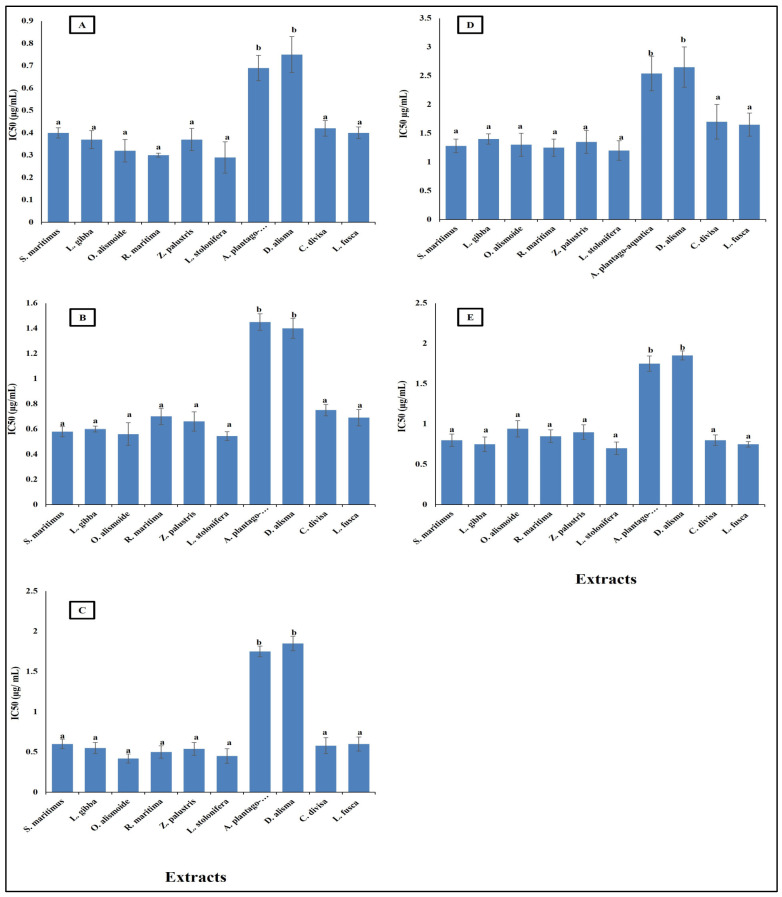
Antiparasitic activity of different aquatic plant extracts against different parasites [(**A**) *O. viverrini*, (**B**) *O. felineus*, (**C**) *C. sinensis*, (**D**) *P. falciparum*, (**E**) *L. donovani*]. The IC50 value, indicating the concentration needed to inhibit 50% of parasite growth. Crude extracts with IC_50_ < 20 µg/mL can be considered as antiparasitic agents [64]. Column value is the mean of five replicates. The error bars represent standard deviation. Columns followed with different letters are significantly different according to ANOVA test.

Parasitic diseases contribute significantly to the global burden of disease, affecting millions of people worldwide. The rising problem of parasite resistance to the existing antiparasitic drugs makes finding new safe antiparasitic agents an urgent need [60]. Plant extracts and other natural products are promising as effective antiparasitic agents [61,62]. The results in Figure 3 show that aquatics had high antiparasitic activity against *Opisthorchis viverrini*, *Opisthorchis. felineus*, *Clonorchis sinensis*, *Plasmodium falciparum*, and *Leishmania donovani* with low IC_50_ (range between 0.7 and 2.5 µg/mL). The lowest antiparasitic activity was recorded in *A. plantago-aquatica* and *D. alisma* with the highest IC_50_, the other extracts’ antiparasitic activities were not significantly different from each other, but they were significantly higher than that of *A. plantago-aquatica* and *D. alisma* with lower IC_50_ (range between 0.3 and 1.7 µg/mL).

This low IC_50_ showed the effectiveness of the aquatic extracts as antiparasitic agents. The differences between the studied aquatic plants in their antiparasitic activity is related to the differences in their phytochemical components, as the extracts lowest in phytochemical content were the lowest in antiparasitic activity. This is in the same line as reported in Ali and Mishra [63], where differences in the phytochemical components in each extract led to different biological activities. The low IC_50_ of the studied aquatic extracts gives them promising antiparasitic activity, as it was reported that crude extracts with IC_50_ < 20 µg/mL can be considered as antiparasitic agents [64]. The high antiparasitic activity of the aquatic plants was related to their high content of secondary metabolites such as phenols, flavonoids, alkaloids, tannins, saponines, and steroids [62]. Similarly, Ponomarev et al. [65] reported that the anti-*Opisthorchis felineus* effects of plant-origin materials were more effective than synthetic drugs. Gupta et al. [60] suggested that the future of antiparasitic agents is in natural products.

### 2.3. Antibacterial Activity of the Extracts

**Figure 4:**
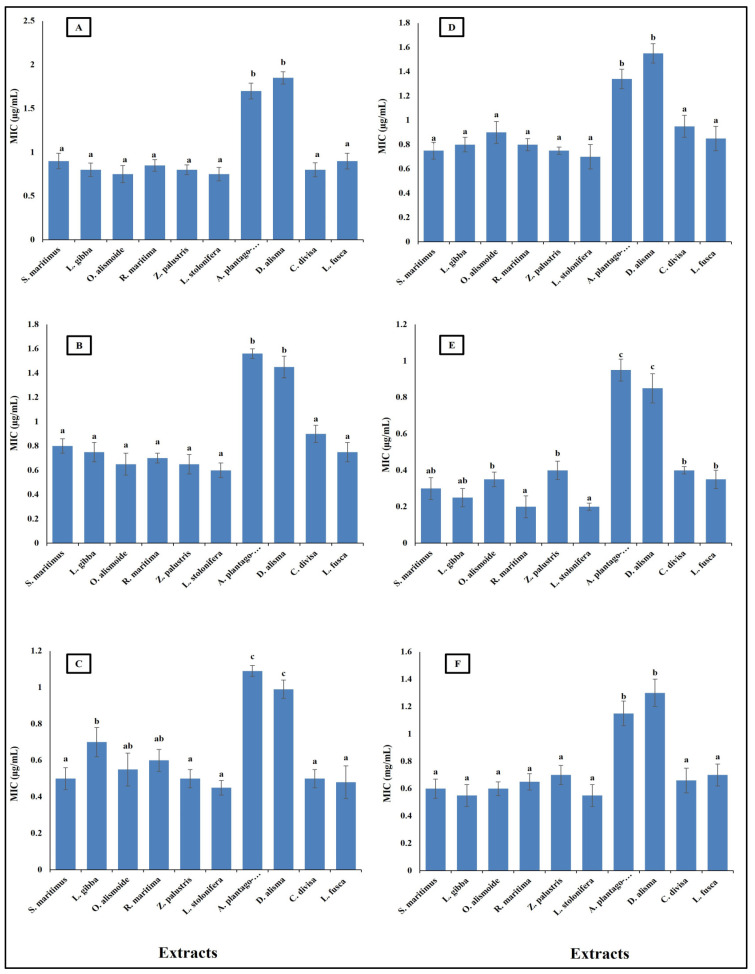
Antibacterial activity of different aquatic plant extracts against different pathogenic bacteria expressed as MIC, which is the lowest concentration of the assayed agent that inhibits microbial growth. [(**A**) *Bacteroides fragilis*, (**B**) *Helicobacter pylori*, (**C**) *Fusobacterium nucleatum*, (**D**) *Porphyromonas gingivalis*, (**E**) *Salmonella enterica*, (**F**) *Neisseria gonorrhoeae*]. Plant crude extracts with MIC lower than 8 µg/mL are outstanding antibacterial agents [62]. Column value is the mean of five replicates. The error bars represent standard deviation. Columns followed by different letters are significantly different according to ANOVA test.

Antibiotic resistance poses a critical challenge to modern medicine, necessitating coordinated efforts across healthcare, research, policy, and public education to mitigate its impact and preserve the efficacy of existing antibiotics. New natural and effective antibacterial agents are greatly needed. Aquatic plants have diverse biological active components, such as phenols, alkaloids, flavonoids, saponines, and tannins; thus, these plants are a promising source for safe and effective antibacterial agents [36]. The results in Figure 4 show the antibacterial activity of different aquatic plant extracts against different pathogenic bacteria, in terms of their minimum inhibitory concentration (MIC) (µg/mL). The studied aquatic plants extracts showed a low MIC. *Alisma plantago-aquatica* and *Damasonium alisma* extracts showed the highest MIC. This was from 0.9 µg/mL against *Salmonella enterica* to 1.8 µg/mL against *Bacteroides fragilis*. The other studied aquatic plants extracts had an MIC lower than unity against the different studied pathogenic bacteria. This low MIC of the extracts makes the studied aquatic plants highly promising antibacterial agents, as it was reported that plant crude extracts with an MIC lower than 8 µg/mL are outstanding antibacterial agents [66]. The different MICs of the extracts are related to the differences in their phytochemicals components. Alzandi et al. [67] reported that plant extracts with different phytochemical components have different antibacterial activity.

**Figure 5:**
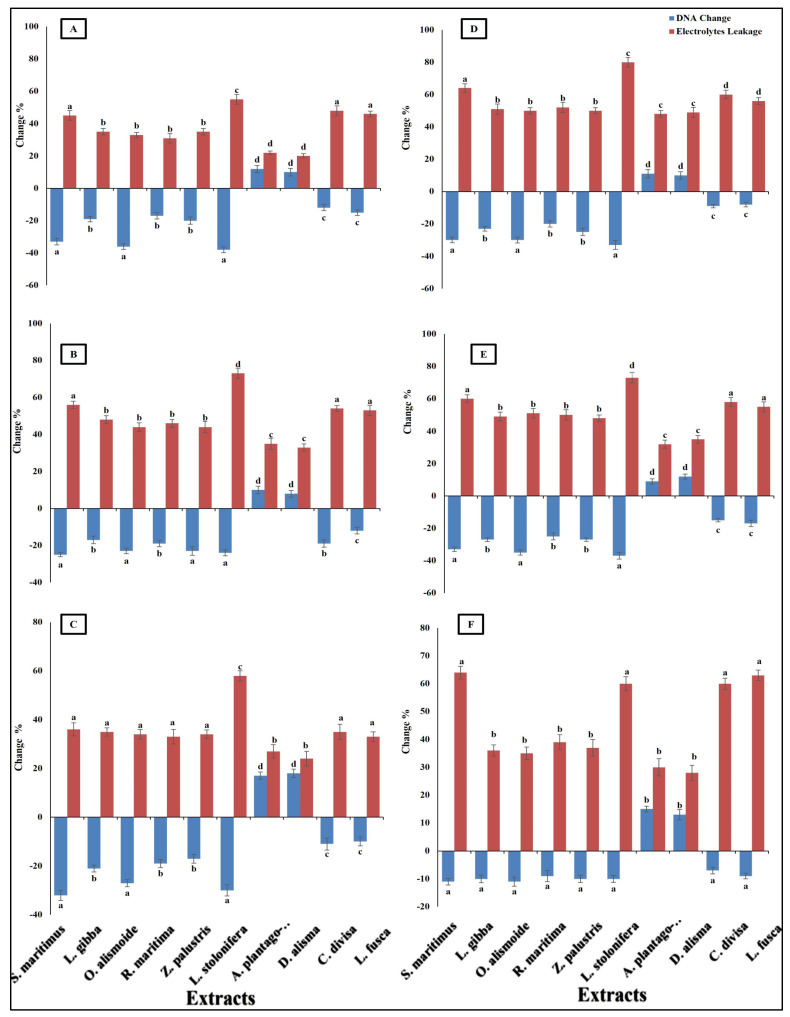
Change in DNA and electrolyte leakage (%) in bacterial species [(**A**) *Bacteroides fragilis*, (**B**) *Helicobacter pylori*, (**C**) *Fusobacterium nucleatum*, (**D**) *Porphyromonas gingivalis*, (**E**) *Salmonella enterica*, (**F**) *Neisseria gonorrhoeae*] after treatment, with MIC of each extract. The negative value of the changes in DNA clarified that the DNA in the treated bacterial isolates was lower than that in the non-treated isolates, indicating that there was destruction of DNA. Column value is the mean of five replicates. The error bars represent standard deviation. Columns followed by different letters are significantly different according to ANOVA test.

Differences in antibacterial activity against different pathogenic bacteria are related to the different mechanisms through which the antibacterial agent can kill bacterial cells. Antibacterial agents can disturb bacterial cell wall synthesis and plasma membrane permeability. In addition, antibacterial agents can destroy the protein and nucleic acid of bacterial cells [68]. The prepared extracts of aquatic plants showed antibacterial activity through the destruction of DNA (the negative value of the changes in DNA clarified that the DNA in the treated bacterial isolates was lower than that in the non-treated isolates, and this indicated that there had been destruction of the DNA) and an increase in plasma membrane permeability (Figure 5). The results showed that the *Ludwigia stolonifera* extract was the most effective antibacterial agent and the most effective in the destruction of DNA and in increasing the bacterial membrane permeability. The effect of *L. stolonifera* extract on DNA change and electrolyte leakage was followed by *S. maritimus*, *O. alismoide*, *L. gibba, R.maritimac*, and *Z. palustris*. Similarly, Shawky et al. [69] reported the promising antibacterial activity of the genus *Ludwigia*. The lowest effect was recorded in *A. plantago*-aquatica and *D. alisma.* The change in DNA had a positive value that indicated that there was replication in the DNA and no effective destruction in the DNA, as the treated isolates had a DNA content higher than that of the non-treated isolates. In addition, these two extracts showed the lowest electrolyte leakage, which means the lowest adverse effect on the bacterial plasma membrane permeability. The differences in the effects between the studied extracts was due to their different phytochemical components [67].

**Figure 6:**
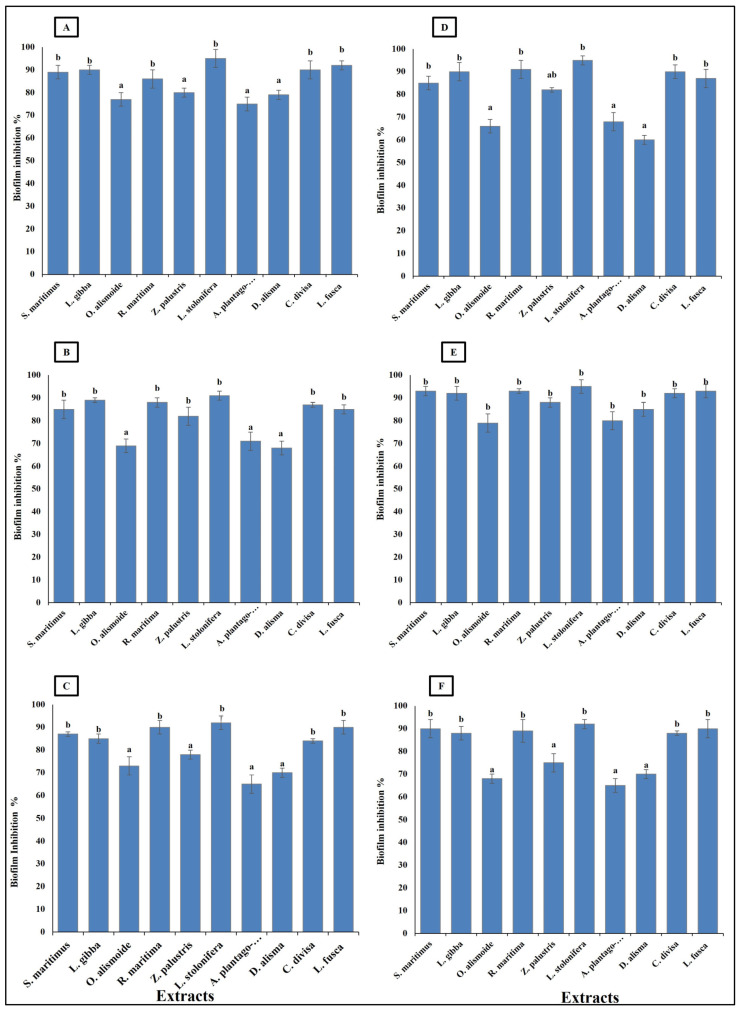
Biofilm inhibition (%) in bacterial species [(**A**) *Bacteroides fragilis*, (**B**) *Helicobacter pylori*, (**C**) *Fusobacterium nucleatum*, (**D**) *Porphyromonas gingivalis*, (**E**) *Salmonella enterica*, (**F**) *Neisseria gonorrhoeae*], after treatment, with MIC of each extract. Column value is the mean of five replicates. The error bars represent standard deviation. Columns followed by different letters are significantly different according to ANOVA test.

*Bacteroides fragilis*, *Fusobacterium nucleatum*, *Helicobacter pylori*, *Porphyromonas gingivalis*, *Neisseria gonorrhoeae*, and *Salmonella enterica* are considered carcinogenic bacteria, as persistence infection without control is highly related to gastrointestinal cancer [70]. These bacterial species are known for their ability to form biofilms, which enables them to persist in the infection site, causing serious health problems. Thus, it is important that antibacterial agents show biofilm inhibition activity [71]. The studied aquatic plants extract showed the ability to inhibit the formation of bacterial biofilm. The ability of the extracts to inhibit the bacterial biofilm formation was related to their phytochemical components, as the highest phytochemical components (*Ludwigia stolonifera* extract) showed the highest biofilm inhibition ability, with an inhibition percentage more than 90%. On the other hand, the lowest phytochemical components (*Alisma plantago*-aquatica, and *Damasonium alisma* extracts) showed the lowest biofilm inhibition ability, with an inhibition percentage from 60 to 80% (Figure 6). Similarly, Pallavi et al. [72] reported that the anti-biofilm and anti- adherence potential of plant extracts depends on their phytochemical components and active components, which inhibit the secretion of the bacterial extracellular components used in bacterial biofilm formation. Phenols have the ability to inhibit biofilm formation through disruption of the expression of the genes involved in virulence and adherence [73]. Sehgal et al. [74] reported that extracts with hydrolyzed tannins (low tannin content) could not inhibit bacterial biofilm formation, but extracts with high tannin content had the ability to inhibit 90% of bacterial biofilm formation.

### 2.4. Anticancer Activity of the Extracts

**Figure 7:**
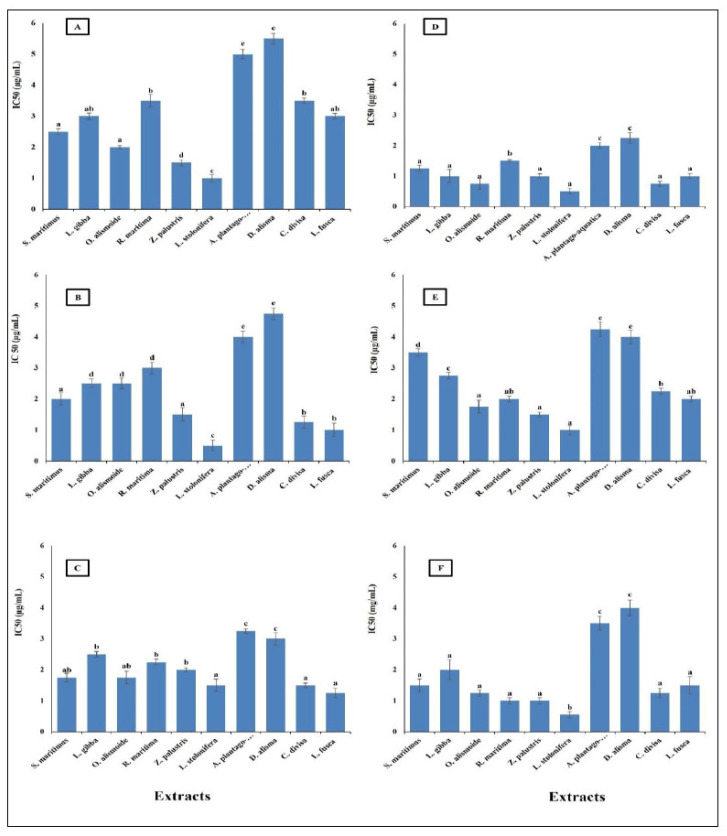
Anticancer activity of different aquatic plant extracts on different human cancer cell lines. (**A**) Gasteric cancer (CLS-145), (**B**) Pancreatic cancer (AsPC-1), (**C**) Liver cancer (HepG2), (**D**) Esophagus cancer (KYSE-410), (**E**) Breast cancer (MCF-7), (**F**) Colon cancer (HCT116). The American National Cancer Institute (NCI) guidelines reported that a crude extract can be considered an anticancer agent when its IC_50_ is less than 30 μg/mL [74]. Column value is the mean of five replicates. The error bars represent standard deviation. Columns followed by different letters are significantly different according to ANOVA test.

Cancer is a significant global health problem that poses severe challenges to healthcare systems worldwide. It is characterized by the uncontrolled growth and spread of abnormal cells, which can invade and damage surrounding tissues and organs [75]. Cancer is one of the leading causes of death globally. According to the World Health Organization (WHO), there were approximately 19.3 million new cases and 10 million cancer deaths in 2020 [76]. The most common cancers include breast, lung, and the different gastrointestinal cancers [77]. Finding new safe effective anticancer agents has become an urgent need [76]. The results in Figure 7 show that the studied aquatic plants extract showed promising anticancer activity against the different cancer cell lines tested. The highest anticancer activity was recorded in the *L. stolonifera* extract, with the lowest IC_50_ range from 0.5 µg/mL against pancreatic cancer cell line (AsPC-1), esophagus cancer cell line (KYSE-410), and colon cancer cell line (HCT116) to 1.5 µg/mL against gasteric cancer cell line (ClS-145). The lowest anticancer activity was in *D. alisma* with the highest IC_50_, which ranged from 2.25 µg/mL against esophagus cancer cell line (KYSE-410) to 5.5 µg/mL against gasteric cancer cell line (ClS-145). In general, the IC_50_ of the prepared extracts was lower than 6 µg/mL against the different cancer cell lines. This low IC_50_ is considered very promising for anticancer agents, as the American National Cancer Institute (NCI) guidelines reported that a crude extract can be considered an anticancer agent when its IC_50_ is less than 30 μg/mL [78]. Similarly, Jamaludin et al. [79] reported the effective anticancer activity of certain marine sponges with an IC_50_ lower than 30 µg/mL. The difference in the anticancer activity between the extracts was related to the difference in their phytochemical components. Huang et al. [80] reported a relationship between the anticancer activity of plant extracts and their phytochemical compositions.

**Figure 8:**
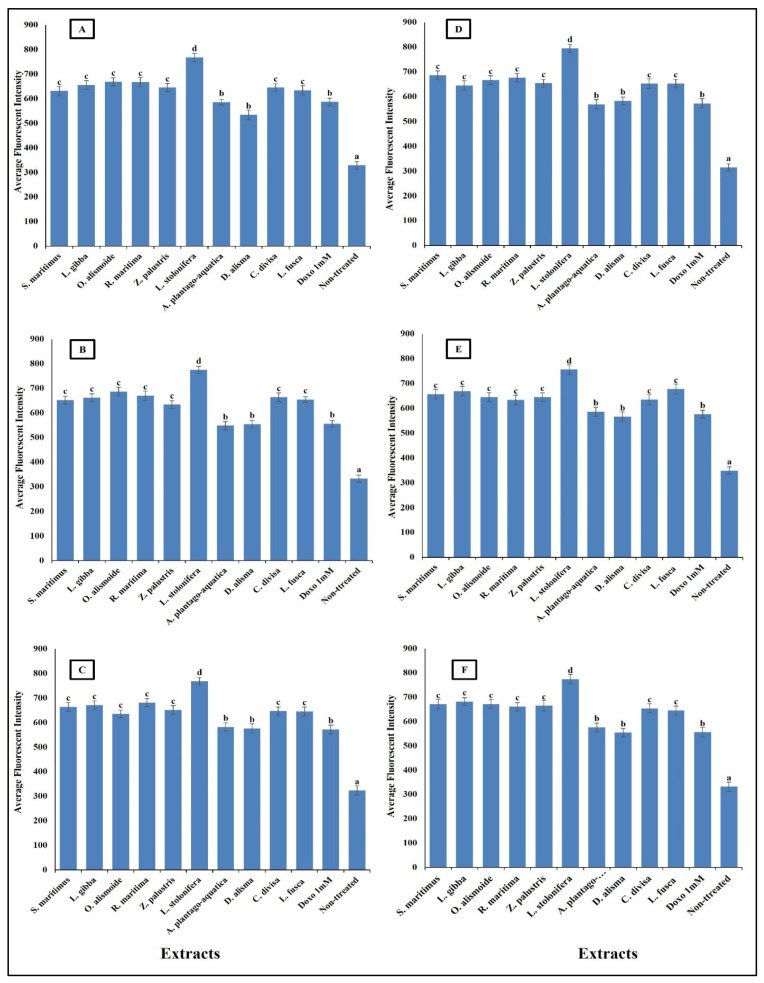
Effect of IC_50_ of different aquatic plant extracts and Doxo 1 mM on nuclear intensity of different human cancer cells. (**A**) Gasteric cancer (CLS-145), (**B**) Pancreatic cancer (AsPC-1), (**C**) Liver cancer (HepG2), (**D**) Esophagus cancer (KYSE-410), (**E**) Breast cancer (MCF-7), (**F**) Colon cancer (HCT116). Nuclear intensity refers to the mean fluorescence intensity measured within the nuclear region of cells stained with Hoechst 33258 (λ ex = 352 nm, λ_em = 461 nm). Visualization was conducted using a Cellomics ArrayScan HCS reader (Thermo Scientific, Waltham, MA, USA), and quantification of fluorescence intensity was performed using a Cell Health Profiling bioapplication module. The relationship between nuclear intensity and average fluorescence intensity is directly proportional, providing insights into the distribution and localization of the fluorescent marker within the cells. Column value is the mean of five replicates. The error bars represent standard deviation. Columns followed by different letters are significantly different according to ANOVA test.

**Figure 9:**
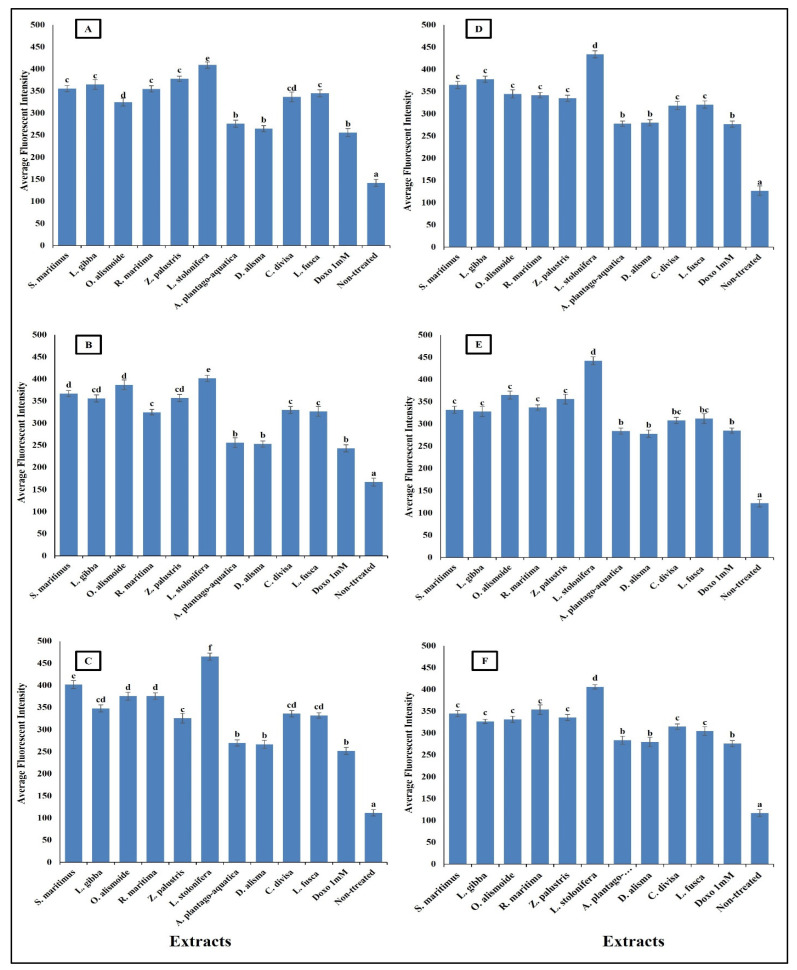
Effect of IC_50_ of different aquatic plant extracts and Doxo 1 mM on plasma membrane permeability of different human cancer cells. (**A**) Gasteric cancer (CLS-145), (**B**) Pancreatic cancer (AsPC-1), (**C**) Liver cancer (HepG2), (**D**) Esophagus cancer (KYSE-410), (**E**) Breast cancer (MCF-7), (**F**) Colon cancer (HCT116). Plasma membrane permeability refers to the ability of the plasma membrane to allow certain molecules or ions to pass through it by diffusion or active transport mechanisms. The average fluorescence intensity is used as an indicator of plasma membrane permeability. When the plasma membrane becomes more permeable, there is an increased influx or efflux of fluorescent dyes (cell permeability dye (Excitation 491/Emission 509), leading to changes in the average fluorescence intensity measured within the cells. Column value is the mean of five replicates. The error bars represent standard deviation. Columns followed with different letters are significantly different according to ANOVA test.

Anticancer agents affect cancer cells through multiple mechanisms, often targeting specific cellular structures and functions. Thus, to evaluate the mechanism by which the prepared extracts destroyed the cancer cells, we evaluated the change in nuclear intensity, plasma membrane permeability, mitochondrial membrane permeability, and cytochrome C release as a response to the different IC_50_ levels of the prepared extract and 1 mM doxorubicin (positive control) for comparison of their effectiveness. The results in Figure 8 show that nuclear intensity increased under treatment with the aquatic plant extracts or doxorubicin. However, the treatment with aquatic plant extracts caused a higher increase in cancer cell nuclear intensity than doxorubicin. Changes in nuclear intensity revealed a change in the nucleic acids and chromatin, which disturbed the cell cycle [81]. Anticancer agents can alter nuclear intensity by inducing DNA damage, disrupting the cell cycle, and promoting apoptosis. Many anticancer agents cause DNA breaks or cross-links, or interfere with DNA replication and transcription. This leads to increased nuclear intensity, due to DNA condensation during apoptosis [82,83]. Changes in plasma membrane permeability are a hallmark of apoptosis and can be triggered by various anticancer agents [84,85]. The studied aquatic plant extracts increased the plasma membrane permeability of the cancer cells more than doxorubicin (Figure 9). Anticancer agents can activate apoptotic pathways, leading to externalization of phosphatidylserine and increased membrane permeability [86,87]. At higher concentrations, some anticancer drugs may induce necrosis, causing loss of membrane integrity and uncontrolled release of cellular contents [88,89].

**Figure 10:**
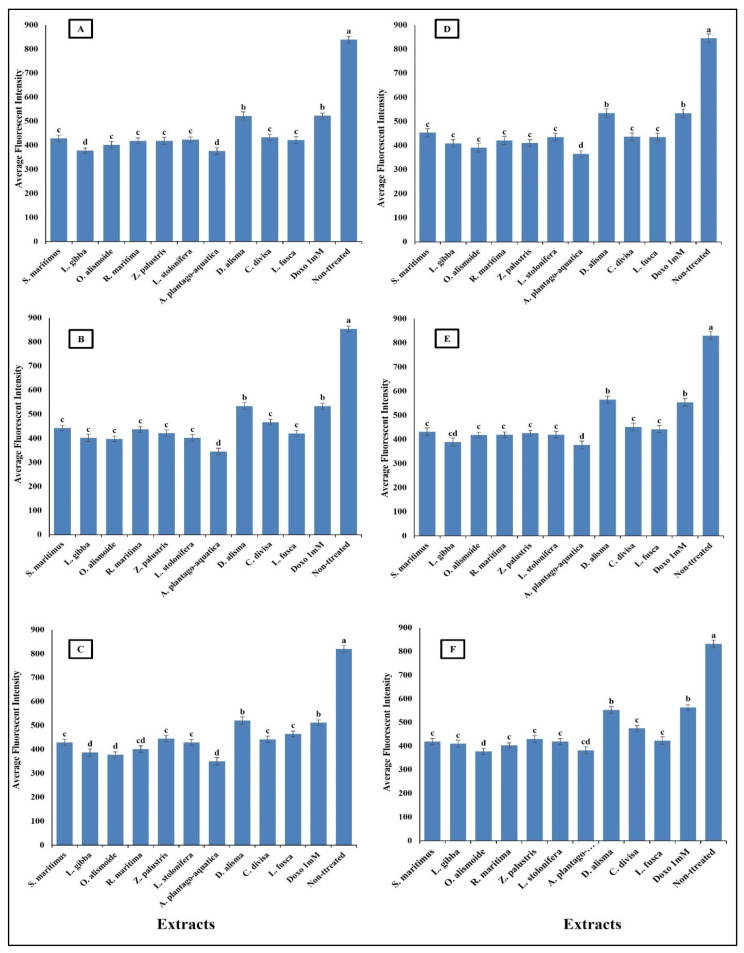
Effect of IC_50_ of different aquatic plant extracts and Doxo 1 mM on mitochondrial membrane permeability (MMP) of different human cancer cells. (**A**) Gasteric cancer (CLS-145), (**B**) Pancreatic cancer (AsPC-1), (**C**) Liver cancer (HepG2), (**D**) Esophagus cancer (KYSE-410), (**E**) Breast cancer (MCF-7), (**F**) Colon cancer (HCT116). MPP measurement was based on the mean intensity of MMP dye (Excitation 552/Emission 576) penetrating the mitochondria; the lower the fluorescent intensity, the higher the effect against the mitochondria. Column value is the mean of five replicates. The error bars represent standard deviation. Columns followed by different letters are significantly different according to ANOVA test.

**Figure 11:**
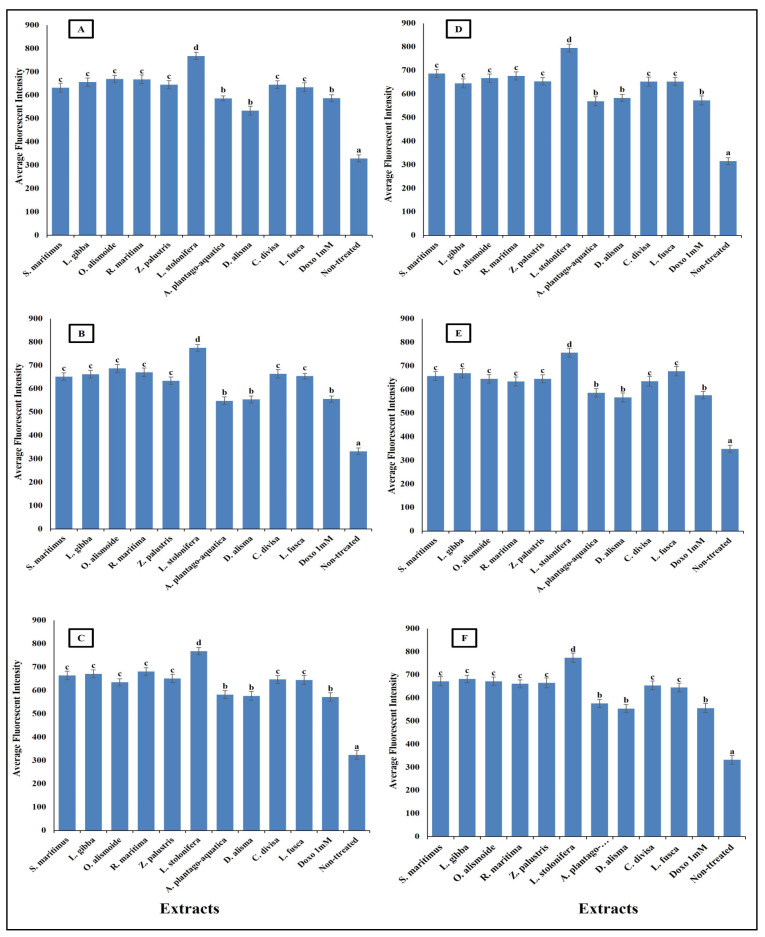
Effect of IC_50_ of different aquatic plant extracts and Doxo 1 mM cytocrome c release in different human cancer cells. (**A**) Gasteric cancer (CLS-145), (**B**) Pancreatic cancer (AsPC-1), (**C**) Liver cancer (HepG2), (**D**) Esophagus cancer (KYSE-410), (**E**) Breast cancer (MCF-7), (**F**) Colon cancer (HCT116). Cytochrome c release from the mitochondria into the cytosol is a key event in the apoptotic pathway. In our study, we quantified cytochrome c release by measuring the average fluorescence intensity of a cytochrome c-specific fluorescent probe. Visualization was conducted using a Cellomics ArrayScan HCS reader (Thermo Scientific), and quantification of fluorescence intensity was performed using a Cell Health Profiling bioapplication module. When cytochrome c is released from the mitochondria, it binds to the fluorescent probe, resulting in an increase in fluorescence intensity. Column value is the mean of five replicates. The error bars represent standard deviation. Columns followed with different letters are significantly different according to ANOVA test.

Mitochondrial membrane permeability (MMP) is an indicator for apoptosis [90]. An MMP dye was used to assess the functionality of active mitochondria, as it accumulates in organelles that maintain inner membrane potential. The measurement of MMP was based on the mean intensity of the dye within the mitochondria; a lower fluorescence intensity indicated a greater negative impact on mitochondrial function and an increase in the MMP [91]. The studied aquatic plant extracts significantly increased the MMP of the different cancer cells (Figure 10). Many anticancer agents increase mitochondrial membrane permeability, leading to mitochondrial dysfunction and apoptosis. Anticancer agents promote the release of pro-apoptotic proteins that permeabilize the outer mitochondrial membrane [92,93]. Increasing MMP leads to cytochrome c release. The release of cytochrome c from mitochondria into the cytosol is a critical step in the intrinsic apoptotic pathway, often induced by anticancer agents [94]. The results in Figure 11 clarify the significant increase in cytochrome c release after treatment with the prepared aquatic plant extracts and doxorubicin, but the cytochrome c release was the highest in the case of aquatic plant extract treatment. Once in the cytosol, cytochrome c binds to Apaf-1, leading to the formation of apoptosome and activation of caspase-9, which subsequently activates caspase-3 and other executioner caspases, triggering a cascade of proteolytic events that lead to the orderly dismantling of the cell. This cascade ensures that apoptosis proceeds efficiently, allowing for the removal of damaged or unwanted cells without eliciting an inflammatory response [95,96,97,98].

Interestingly, the plant crude extracts possessed a considerably higher activity than the pure standard bioactive compound (Doxorubicin). Crude extracts can sometimes be more effective than pure standard anticancer agents. This can be due to the presence of a mixture of compounds in the crude extracts that can work together synergistically to enhance the overall anticancer effect [99]. These compounds may act on different targets or pathways, providing a broader and more effective response. While pure compounds typically target a specific molecule or pathway, crude extracts may affect multiple targets simultaneously. This can be beneficial in cancer treatment, where multiple pathways are often dysregulated [100]. Cancer cells can develop resistance to single-agent therapies over time. The complex mixture of compounds in crude extracts may reduce the likelihood of the development of resistance [101,102].

## 3. Materials and Methods

### 3.1. Aquatic Plants Extract Preparation

Shoot systems of *Scirpus maritimus*, *Lemna gibba*, *Otellia alismoide*, *Ruppia maritima*, *Zannichellia palustris*, *Ludwigia stolonifera*, *Alisma plantago-aquatica*, *Damasonium alisma*, *Carex divisa*, and *Leptochloa fusca* were collected from various branched canals of the Nile River in Egypt, Sharqia Governorate (30.7° N 31.63° E), in the period from January 2023 to June 2023. Plants were identified according to the local people and Zahran [37]. After drying, the shoot systems were finely ground, and aqueous extracts were prepared following the method of Naguib and Tantawy [103]. The ground powder (100 g) was extracted in 50 mL dist. water for 24 h on an orbital shaker (100 rpm) at 30 °C. After incubation, the mixture was filtrated through Whitman filter paper No. 1, the filtrate was dried, and designated as the plant extracts.

### 3.2. Phytochemical Components and Antioxidant Capacity of the Extracts

The antioxidant capacity and phytochemical composition of the extracts were evaluated through various assays.

The total antioxidant capacity of different concentrations (50, 100, and 150 mg/mL) of prepared extracts was determined in comparison with a standard (Vitamin C) using a DPPH radical scavenging assay, following Blois [104]. The reaction mixture consisted of 1.6 mL of prepared extract (with different concentrations) and 2.4 mL of 0.1 mM DPPH, shaken well and incubated in the dark at room temperature for 30 min. Absorbance was measured at 517 nm after incubation. The percentage of DPPH radical scavenging activity was calculated using the following equation:

%DPPH Radical Scavenging Activity = (Ac − As)/Ac × 100

where Ac is the absorbance of the control and As is the absorbance of the sample.

Phytochemical components including tannins, saponins, steroids, alkaloids, flavonoids, and phenols were quantified following methods described by Harbourne [105] with adjustments by Trease and Evans [106] for tannin, saponin, steroid, and alkaloid contents. Total flavonoid content was measured as per Pallab et al. [107], using an aluminum chloride colorimetric assay, while total phenol content was determined according to Julkunen-Tiitto [108].

Tannin content was measured by adding 0.3 mL of 0.1 N FeCl3 in 0.1 N HCl (3 mL) to 2 mL of plant extracts, along with 0.3 mL of 0.0008 M potassium ferricyanide. The absorbance was recorded at 720 nm, and tannin concentration was calculated using a standard curve.

Steroid content was measured by adding cholesterol color reagent to plant extracts and incubating the mixture for 35 min at room temperature. Absorbance was recorded at 550 nm, and steroid concentration was determined using a cholesterol standard curve.

Saponin content was measured by evaporating 0.5 mL of plant extract to dryness, then adding a fresh solution of vanillin acetic acid and perchloric acid. After incubation, the absorbance was recorded at 550 nm, and saponin concentration was determined using a sapogenin standard curve.

Alkaloid content was measured by adding 60% H_2_SO_4_ to the plant extract and incubating the mixture at room temperature for 3 h. Absorbance was recorded at 565 nm, and alkaloid concentration was calculated using an atropine standard curve.

Total flavonoid content was measured by incubating the plant extract with a sodium nitrite solution, followed by the addition of aluminum chloride and NaOH. Absorbance was recorded at 510 nm, and total flavonoid content was calculated using a quercetin standard curve.

Total phenol content was measured by mixing the plant extract with Folin–Ciocalteu reagent and Na_2_CO_3_. After incubation, absorbance was recorded at 725 nm, and phenol concentration was determined using a pyrogallol standard curve.

### 3.3. Antiparasitic Activity

Adult worms of *Opisthorchis viverrini*, *Opisthorchis felineus*, and *Clonorchis sinensis* were cultured at 37 °C for 24 h in RPMI 1640 medium containing 1% glucose, 0.1 mg/mL streptomycin, and penicillin, within a CO_2_ incubator. To calculate the IC_50_, various concentrations of extracts were introduced into the medium, and both treated and untreated cultures were then cultured for an additional 24 h. The viability of the worms was examined under an inverted microscope, and IC_50_ values were determined using a dose–response curve according to the method described by Pakharukova et al. [109].

Different concentrations of extracts were assessed for their antimalarial activity in vitro against the chloroquine-resistant FCB1 strain of *Plasmodium falciparum*, with the IC_50_ being determined as a measure of activity [110]. In a standard in vitro assay for antimalarial activity, *Plasmodium falciparum* cultures were synchronized and incubated with various concentrations of plant extracts or compounds in a 96-well plate at 37 °C for 48 h. Parasite growth was assessed using microscopy to determine parasitemia. The percentage inhibition of parasite growth was calculated by comparing treated samples to controls, and the IC50 value, indicating the concentration needed to inhibit 50% of parasite growth, was determined to evaluate the efficacy of the test samples.

Regarding leishmanicidal activity in vitro, *Leishmania donovani* was employed, and the antileishmanial screening followed the protocol outlined by Mbongo et al. [111]. In an in vitro assay for leishmanicidal activity, *Leishmania donovani* were cultured with various concentrations of plant extracts and incubated in a 96-well plate at 26 °C for 72 h. Parasite viability was assessed using direct counting under a microscope after staining. The percentage inhibition of parasite growth was calculated by comparing treated samples to controls; and the IC50 value, indicating the concentration needed to inhibit 50% of parasite growth, was determined to evaluate the efficacy of the test samples.

### 3.4. Antibacterial Activity

#### 3.4.1. Pathogenic Bacterial Strains

Pathogenic Gram negative bacteria *Bacteroides fragilis* (ATCC 29762), *Fusobacterium nucleatum* (ATCC 25586), *Helicobacter pylori* (ATCC 51407), *Porphyromonas gingivalis* (ATCC 33277), *Neisseria gonorrhoeae* (ATCC 43070), and *Salmonella enterica* (ATCC 14028) were obtained from the American Type Culture Collection (Manassas, VA, USA).

#### 3.4.2. Determination the Minimal Inhibitory Concentration (MIC) of Each Extract against Different Pathogenic Bacteria

The MIC of dried extracts was evaluated through a broth dilution assay. Extracts were dissolved, serially diluted in brain heart infusion broth, inoculated with bacteria, and incubated. Following incubation, turbidity was assessed at OD 600 nm. MIC was the lowest concentration of the studied extract that inhibited the microbial growth [112].

#### 3.4.3. Antibacterial Mechanism

##### Determination of Bacterial Cell Membrane Permeability

Permeability changes were expressed as the percentage of relative electric conductivity [113]. After culturing for 12 h at 37 °C, bacteria were centrifuged at 1500× *g* for 10 min. Cells were washed with 5% glucose until their electric conductivities approximated that of 5% glucose, defining them as isotonic bacteria. The electric conductivities of the prepared aquatic extracts were evaluated after adding 5% glucose and the MIC of each extract (designated as *A*). Isotonic bacteria were then incubated with the MIC of each extract at 37 °C for 12 h, after which, conductivities were measured and recorded (designated as *B*). For reference, the conductivity of bacteria in 5% glucose was treated for 5 s in boiling water and noted as (*C*). The relative electric conductivity was calculated using the following equation:

$$Relative\;electric\;conductivity\;\left(\%\right)=\frac{B-A}{C}\times 100$$

##### Determination of Changes in Bacterial DNA Content

Bacterial suspension with OD600 nm = 2.0 was treated with the MIC of extracts at 37 °C for 12 h, and bacterial precipitation was obtained by refrigerated centrifugation (10,000× *g*, 1 min). Bacterial genomic DNA extraction kits (Tian Gen Biotech Co., Ltd., Beijing, China) were utilized to determine intracellular DNA content. The change in bacterial DNA content was calculated using the following equation:

$$Change\;in\;DNA\;\left(\%\right)=\frac{DNA\;in\;the\;treated\;isolate-DNA\;in\;the\;untreated\;isolate}{DNA\;in\;the\;untreated\;isolate}\times 100$$

#### 3.4.4. Anti-Biofilm Activity

The effectiveness of aquatic extracts in inhibiting biofilm formation was evaluated using the crystal violet staining technique. Biofilms cultivated in 96-well flat-bottom polystyrene plates were exposed to the MIC of the extracts and incubated at 37 °C for 24 h. Following incubation, each plate was rinsed with distilled water to remove any planktonic cells and air-dried. Subsequently, 125 µL of 0.1% crystal violet dye was added to stain the adherent biofilm. The biofilm was dissolved by adding 130 µL of 30% acetic acid, and the absorbance of each plate was measured at 550 nm using a Microplate absorbance reader to assess biofilm inhibition [114].

### 3.5. Anticancer Activity

#### 3.5.1. Cell Viability Assay

Various cancer cell lines (gastric cancer cell line (CLS-145), pancreatic cancer cell line (AsPC-1), liver cancer cell line (HepG2), colon cancer cell line (HCT116), esophagus cancer cell line (KYSE-410), and breast cancer cell line (MCF-7) from Cell Line Service (Eppelheim, Germany) were obtained and cultured. The 3-(4,5-dimethyl-2-thiazolyl)-2,5-diphenyl-2H-tetrazolium bromide (MTT) method was employed for cell viability assays. In this assay, cells were incubated with the yellow tetrazolium salt (MTT). Viable cells with active metabolism reduced MTT to purple formazan crystals. After incubation, the formazan crystals were solubilized using DMSO. The absorbance of the solution was then measured at 570 nm using a spectrophotometer. The intensity of the color correlated with the number of viable cells, allowing for the determination of the cell viability and cytotoxicity of test compounds.

#### 3.5.2. High-Content Screening Assay

A high-content screening (HCS) assay was conducted to assess toxicity on the different studied cancer cells, examining various parameters such as nuclear intensity, membrane permeability, mitochondria membrane permeability, and cytochrome c. Cancer cells (10^5^) were seeded in twelve-well plates and incubated at 37 °C with 5% CO_2_ for 24 h. Subsequently, cells were treated with IC_50_ concentrations of different prepared extracts, alongside untreated cells (negative control) and cells treated with 1 mM doxorubicin (positive control). After another 24 h incubation period, MMP dye (Excitation 552/Emission 576) and cell permeability dye (Excitation 491/Emission 509) were applied to living cells, followed by a 1 h incubation. Fixation (4% formaldehyde, 15 min) and permeabilization (0.1% Triton X-100 in PBS) were then carried out, followed by blocking with 3% bovine serum albumin and incubation with cytochrome c primary mouse antibody for 1 h. Samples were washed three times with wash buffer I (1:6 PBS), followed by addition of goat anti-mouse secondary antibodies conjugated with DyLightTM 649. Cells were rinsed with wash buffer II (1:6 PBS with 1% Tween-20) and stained with Hoechst 33258 (λ ex = 352 nm, λ_em = 461 nm) to visualize nuclei. Visualization was conducted using a Cellomics ArrayScan HCS reader (Thermo Scientific). Quantification of the fluorescence intensity of each dye, to examine parameters such as nuclear intensity, membrane permeability, mitochondria membrane permeability, and cytochrome c, was performed using a Cell health profiling bioapplication module [115].

Nuclear intensity was determined as the mean fluorescence intensity measured within the nuclear region of cells stained with Hoechst 33258. The relationship between nuclear intensity and average fluorescence intensity is directly proportional, i.e., an increase in nuclear intensity leads to an increase in fluorescence intensity. This provided insights into the distribution and localization of the fluorescent marker within the cells.

Plasma membrane permeability refers to the ability of the plasma membrane to allow certain molecules or ions to pass through it by diffusion or active transport mechanisms. The average fluorescence intensity is used as an indicator of plasma membrane permeability. When the plasma membrane becomes more permeable, there is an increased influx or efflux of fluorescent dyes (cell permeability dye (Excitation 491/Emission 509), leading to changes in the average fluorescence intensity measured within the cells.

MPP measurement was based on the mean intensity of MMP dye penetrating the mitochondria; the lower the fluorescent intensity, the higher the effect against the mitochondria.

Cytochrome c release from the mitochondria into the cytosol is a key event in the apoptotic pathway. In our study, we quantified cytochrome c release by measuring the average fluorescence intensity of a cytochrome c-specific fluorescent probe. When cytochrome c is released from the mitochondria, it binds to the fluorescent probe, resulting in an increase in fluorescence intensity.

### 3.6. Statistical Analysis

Data analysis was conducted using SPSS software (version 14), with results expressed as mean ± SD. An ANOVA test was used for comparing mean values.

## 4. Conclusions

Aquatic plants are a rich source of pharmaceutical compounds such as phenols, tannins, saponins, alkaloids, and flavonoids. Among the studied plants, *Ludwigia stolonifera* exhibited the highest levels of phytochemicals, followed by *Scirpus maritimus*, *Lemna gibba*, *Ottelia alismoides*, *Ruppia maritima*, *Zannichellia palustris*, *Carex divisa*, and *Leptochloa fusca*. The lowest levels were found in Alisma plantago-aquatica and Damasonium alisma. Extracts from these plants’ shoots showed significant antioxidant, antibacterial, antiparasitic, and anticancer activities, surpassing the antioxidant capacity of vitamin C. The extracts had IC_50_ values for antiparasitic activity ranging from 0.7 to 2.5 µg/mL and demonstrated low minimum inhibitory concentrations (MIC) against various pathogenic bacteria, causing DNA damage, increased plasma membrane permeability, and 90% biofilm inhibition. They were also effective against a range of cancer cell lines, with IC50 values below 6 µg/mL, indicating significant apoptotic activity, increased nuclear intensity, plasma membrane permeability, mitochondrial membrane permeability, and cytochrome c release, and outperforming doxorubicin. This study underscores the potential of aquatic plants as sources of new, safe, and effective drugs with strong antiparasitic, antibacterial, and anticancer properties. Future research should focus on identifying specific pharmaceutical compounds in each plant and conducting in vivo studies to compare these extracts with standard medications.

## Data Availability

All data generated in this study are found in the manuscript.

## Supplementary Materials

The following supporting information can be downloaded at: https://www.mdpi.com/article/10.3390/plants13152148/s1, Figure S1: Studied aquatic plants; Table S1: English name, scientific name, classification, and plant morphological characters.
