# Supplementary material for: Phytochemical Composition and Bioactivities of Some Hydrophytes: Antioxidant, Antiparasitic, Antibacterial, and Anticancer Properties and Mechanisms
Source: Plants (Basel). 2024 Aug 2;13(15):2148. doi: 10.3390/plants13152148 (PMC11313917; doi:10.3390/plants13152148)
Supplement: Supplementary file 1 [file plants-13-02148-s001.zip › plants-3043871-supplementary.pdf]

Table S1: English name, scientific name, classification, and plant morphological characters

English name	Scientific name	Classification	Morphological characters
Sea Club-rush	*Scirpus maritimus*	**Family:** Cyperaceae<br>**Order:** Poales	• Perennial, rhizomatous herb<br>• Stems are triangular in cross-section, up to 1.5 meters tall<br>• Leaves are long, narrow, and grass-like<br>• Inflorescence is a cluster of spikelets, usually brown
Fat Duckweed	*Lemna gibba*	**Family:** Araceae<br>**Order:** Alismatales	• Small, free-floating aquatic plant<br>• Thalli are oval and usually inflated, giving a bloated appearance<br>• No true stems or leaves, appearing as small, green, oval bodies<br>• Roots are simple, unbranched
Duck Lettuce	*Otellia alismoide*	**Family:** Hydrocharitaceae<br>**Order:** Alismatales	• Submerged aquatic plant<br>• Leaves are long, ribbon-like, and can be floating or submerged<br>• Flowers are white with three petals, emerging above the water surface<br>• Produces floating seeds
Beaked Tasselweed	*Ruppia maritima*	**Family:** Ruppiaceae<br>**Order:** Alismatales	• Submerged, perennial aquatic plant<br>• Thin, flexible stems and narrow, thread-like leaves<br>• Flowers are small, inconspicuous, and wind-pollinated<br>• Produces small, hard fruits
Horned Pondweed	*Zannichellia palustris*	**Family:** Zannichelliaceae<br>**Order:** Alismatales	• Submerged aquatic plant<br>• Slender, branching stems with narrow, thread-like leaves<br>• Flowers are small and unisexual<br>• Fruits are small, horned, and carried by the current
Creeping Water Primrose	*Ludwigia stolonifera*	**Family:** Onagraceae<br>**Order:** Myrtales	• Perennial, creeping aquatic or semi-aquatic herb<br>• Stems are creeping or floating, rooting at the nodes<br>• Leaves are simple, alternate, and ovate to lanceolate<br>• Flowers are yellow with four petals
European Water Plantain	*Alisma plantago-aquatica*	**Family:** Alismataceae<br>**Order:** Alismatales	• Perennial, emergent aquatic plant<br>• Leaves are broad, ovate to lanceolate, and arise from a basal rosette<br>• Flower stems are tall, producing clusters of small, white to pink flowers<br>• Fruits are small, dry, and achene-like
Starfruit	*Damasonium alisma*	**Family:** Alismataceae<br>**Order:** Alismatales	• Emergent, aquatic perennial herb<br>• Leaves are basal, linear to lanceolate<br>• Flower stems are erect, producing small, white to pinkish flowers<br>• Fruits are star-shaped, giving the plant its common name

English name	Scientific name	Classification	Morphological characters
Divided Sedge	*Carex divisa*	**Family:** Cyperaceae<br>**Order:** Poales	• Perennial, clumping sedge<br>• Stems are triangular in cross-section, up to 60 cm tall<br>• Leaves are grass-like, narrow, and arise from the base<br>• Inflorescence is a dense cluster of spikelets, usually brown
Kallar Grass	*Leptochloa fusca*	**Family:** Poaceae<br>**Order:** Poales	• Perennial, tufted grass<br>• Stems are erect, up to 1.5 meters tall<br>• Leaves are linear, flat, and up to 30 cm long<br>• Inflorescence is a loose panicle with slender, spreading branches and spikelets

For more information about plants visit https://www.worldfloraonline.org/

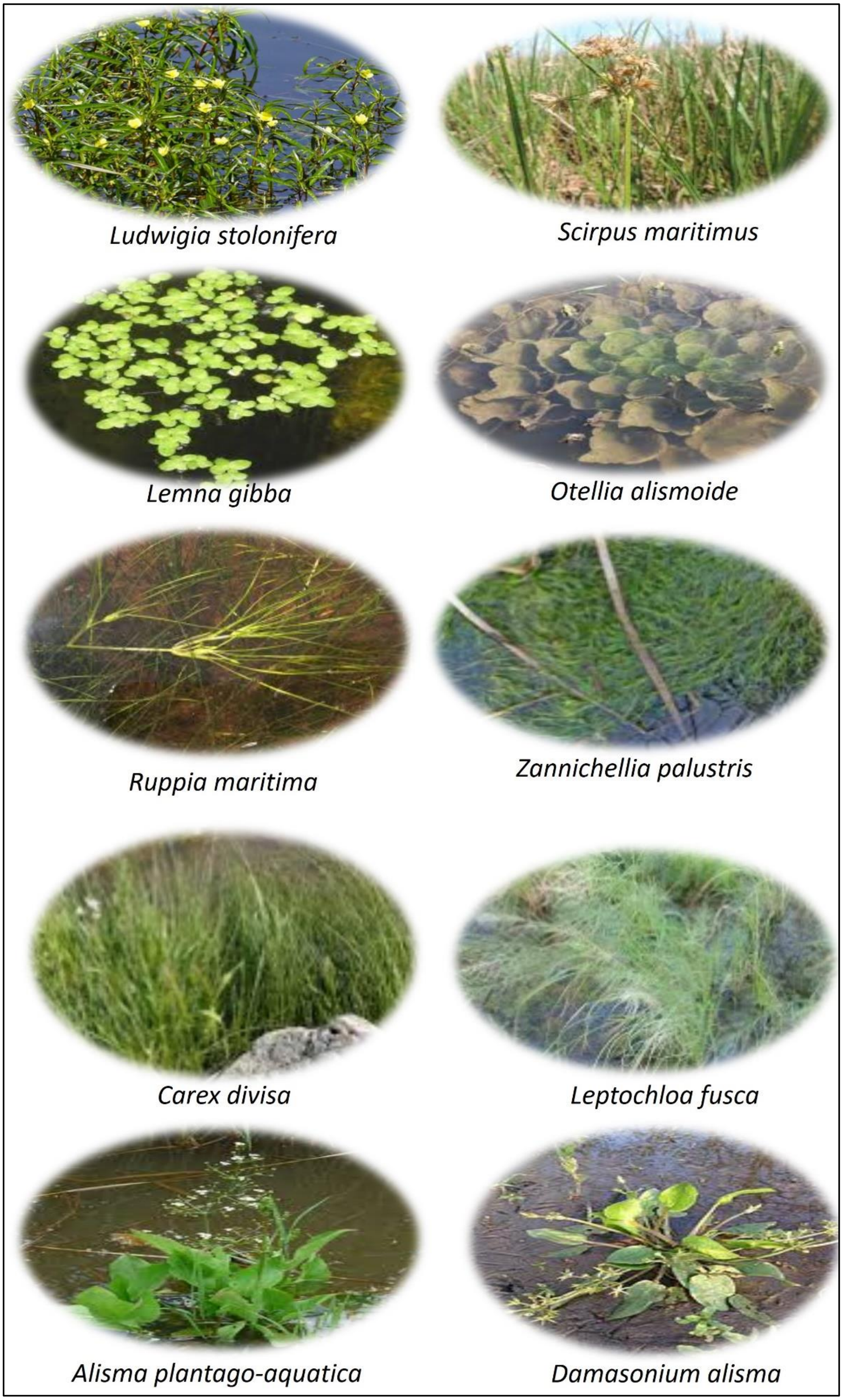

Figure S1: Studied aquatic plants
